# Intersocietal survey on real-world asthma management in Italian children

**DOI:** 10.1186/s13052-024-01718-6

**Published:** 2024-08-01

**Authors:** Maria Angela Tosca, Antonio D’Avino, Giuseppe Di Mauro, Gian Luigi Marseglia, Michele Miraglia del Giudice, Giorgio Ciprandi, SIAIP Delegates, Maddalena Leone, Amelia Licari, Sara Manti. Matteo Naso, Chiara Trincianti, FIMP Delegates, Simona Bellodi, Giancarlo Ottonello, Michele Fiore, Luigi Terracciano, Silvia Zecca

**Affiliations:** 1 Vice President of SIAIP, Allergy Center, IRCCS Istituto Giannina Gaslini, Genoa, Italy; 2 President of FIMP (Italian Federation of Primary Care Pediatricians), Naples, Italy; 3 President of SIPPS, Pediatric Primary Care, National Pediatric Health Care System, Caserta, Italy; 4 Past President of SIAIP, Pediatric Clinic, Fondazione IRCCS Policlinico San Matteo, Pavia, Italy; 5 Department of Clinical, Surgical, Diagnostic and Pediatric Sciences, University of Pavia, Pavia, Italy; 6 President of SIAIP, Department of Woman, Child and General and Specialized Surgery, University of Campania “Luigi Vanvitelli”, Naples, Italy; 7 Allergy Clinic, Casa Di Cura Villa Montallegro, Via Monte Zovetto, 27, Genoa, 16145 Italy

**Keywords:** Asthma, Management, Italy, Primary care, Hospital, Academy

## Abstract

Pediatric asthma management is a compelling challenge for every pediatrician. Different aspects require attention and definition. The present Intersocietal Survey aimed to collect real-world experiences from a sample of Italian pediatricians. A web platform was used to collect anonymous answers to the survey questions.

Four hundred four pediatricians participated in this initiative promoted by the Italian Society of Pediatric Allergy and Immunology (SIAIP), the Society of Preventive and Social Pediatrics (SIPPS), and the Federation of Italian Pediatricians (FIMP).

The results showed an extensive participation of primary care pediatricians (72%). There was a large consensus about diagnostic criteria and medication choice. However, treatment duration and device choice were various. Adherence to guidelines on general aspects of practical clinical management was high.

In conclusion, the present Intersocietal Survey confirmed that pediatric asthma management is rather satisfactory, even if further improvement should concern a more widespread use of ICS for acute asthma/wheezing attacks, a better definition of the duration of ICS and bronchodilator use, and hospital-primary care integration.

## Introduction

Asthma is a prevalent condition in children, with a prevalence of around 10% [1]. Pediatric asthma includes different phenotypes and endotypes. However, type 2 inflammation is prevalent in youth [2, 3].

All children with asthma require careful management, as also mild asthma is at risk of fatal exacerbation, as recently outlined by the Global Initiative for Asthma (GINA) guidelines and a relevant review [4, 5].

The most recent guidelines underline the relevance of asthma control as a goal in managing children with asthma [4]. Despite extensive dissemination of recommendations on the correct and appropriate asthma management, about half of children with asthma have uncontrolled or partially controlled disease [6]. Many factors may cause uncontrolled and severe asthma, including low adherence, incorrect inhalation technique, comorbidity, allergy, infections, pollution, emotional distress (also in the parents), health literacy, low socio-economic situation, and inadequate asthma management [7]. Consequently, pediatric primary care is crucial in managing asthmatic children, and a full hospital-territory integration ensures excellent care. In this regard, the Italian Society of Pediatric Allergy and Immunology (SIAIP) instituted a project on asthma control in children (ControL’Asma Project) to implement asthma management. This project was articulated in the study of various aspects related to asthma control, and it is still ongoing.

A previous survey investigated asthma management in the clinical practice setting, involving 93 Italian primary care pediatricians [8]. These pediatricians were taking care of about 80,000 children. Of these children, 20% had post-infectious wheezing, and 10% had a diagnosis of asthma; less than 2% of asthmatic children had severe asthma. The previous survey also reported that 70% of pediatricians preferred managing children with a pediatric allergist, but 60% directly prescribed the asthma treatment [8]. Eighty % of participants stated that they always assessed the asthma control, and 61% recommended a spacer; 41% of participants always investigated asthma comorbidity, and almost all (97%) pediatricians evaluated the adherence to therapy, even if only 84% always assessed the symptomatic use of salbutamol [8]. In addition, 71% of pediatricians deemed allergen-specific immunotherapy helpful to improve asthma control, but only 20% sent the children to the specialist for its prescription.

However, this survey was carried out before the COVID-19 pandemic. Indeed, this pandemic has profoundly affected health services, but it has also, in a positive sense, emphasized the use of telemedicine and the development of apps. This aspect is enhancing the primary care physician's role as a direct supervisor of patients' health, allowing for more direct and rapid monitoring of clinical conditions without the need for an outpatient visit.

For this reason, the SIAIP Task Force on Asthma promoted a second National Asthma Management Survey. The SIAIP considered it appropriate to involve another scientific societies, namely the Italian Federation of Pediatricians (FIMP) and the Italian Society of Preventive and Social Pediatrics (SIPPS), to increase the initiative's incisiveness and capillarity. Therefore, the present survey aimed to update the previous outcomes concerning pediatric asthma management in Italy.

## Materials and methods

This survey consisted of questions to explore the various aspects of pediatric asthma management in Italy. Three scientific societies (SIAIP, FIMP, and SIPPS) promoted this initiative.

Each Society's institutional website publicized the initiative with a specific banner and a link directly to the survey's web platform. Thus, all Fellows of each society were the target of this initiative.

The SIAIP's organizational secretary (Biomedia, Milan, Italy) provided the platform for anonymous participation.

A panel of independent experts drafted and discussed the questions to be included in the survey.

Table 1 summarizes the list of the questions.

**Table 1:** Questions included in the Intersocietal Survey on the real-world pediatric asthma management in Italy and relevant answers

Questions	Relevant answers
In which region do you work?	Along Italy
Your job position	Primary care 72%; 19% hospital-academy; 9% residents
Years of practice	70% > 15y; 15% 5-15y
Is there a shared hospital-territory care pathway for the management of asthma in your region?	56% yes
What criteria do you use to diagnose wheezing?	96% clinical criteria
How many patients do you follow with recurrent wheezing (at least 3 episodes/year)?	63% < 50; 10% > 100
From what age do you consider a child with recurrent wheezing to be suffering from asthma?	62% from 6 years
When you experience whistling breath (wheezing) do you prescribe inhalation therapy?	99% yes
If you prescribe inhalation therapy, what drugs do you prescribe in acute cases?	ICS + BD 64%; BD 34%
How long on average do you prescribe bronchodilator to treat an acute episode?	51% 6-10 days; 44% 1-5d
How long do you prescribe inhaled corticosteroid in recurrent wheezing or suspected mild persistent asthma?	29% 30-60d; 26% 7-14d; 24% 60-90d
When do you use nebulization as inhalation therapy in children with acute wheezing/asthma episode?	42% patient preference; 29% guidelines; 28% < 3y
When do you use pre-dosed spacer spray as inhalation therapy in children with acute wheezing/asthma?	41% patient preference; 30% guidelines; 29% always; 24% > 3y
On the basis of which criteria do you consider a patient with wheezing to be worthy of diagnostic investigation?	98% clinical; 19% functional; 7% biomarker
Do you think rhinitis contributes to worsening asthma?	85% yes
Do you refer to a hospital referral centre for suspected bronchial asthma?	57% severe asthma attack; 44% difficult management; 40% relapse after ICS discontinuation
Do you provide a written treatment plan of the wheezing/acute asthma attack for the patient to self-manage?	95% yes
Do you recommend early administration of systemic steroids to children who are at greater risk of hospitalisation or more severe forms?	83% yes
Do you periodically review children with asthma and especially after each exacerbation in order to modify the underlying therapy if necessary?	91% yes
What device do you recommend for the child with recurrent wheezing/asthma to perform the background therapy?	60% pre-dose spray with spacer always; 19% > 1y; 18% > 3y
Do you increase background therapy for children who cough or wheeze during sport or other physical exertion?	89% yes
Do you verify at each asthma visit (scheduled or urgent) the correct way of taking therapy using a spacer and pre-dosed spray?	89% yes
Do you have a spacer and spray in your practice to show the patient their correct use?	93% yes
Would you find it useful to have an app for families with the correct way to take medication?	91% yes
Do you recommend specific immunotherapy to patients with asthma or allergic rhinitis?	51% yes; 43% according with referral center

The survey has been conducted from February to April 2024.

The answers and results are reported in Table 1.

## Results

Four hundred four Italian pediatricians completed the survey. The geographic distribution of participants reflected the Italian population. Regarding the job position, 72% were primary care pediatricians, 19% were hospital-university pediatricians, and 9% were residents. Most doctors (70%) had > 15 years of clinical practice. About 80% of participants experienced a shared hospital-territory care integration for managing asthma in their regions. Practically all participants (96%) used clinical criteria to diagnose wheezing. Most participants (63%) cared for < 50 children with recurrent wheezing or asthma, 27% between 50 and 100, and only 10% > 100. Most pediatricians (62%) considered a child with recurrent wheezing to suffer from asthma from 6 years of age, 22% from 3 years, and 15% from 2 years. Almost all participants prescribed inhalation therapy if the child has wheezing; for acute episodes, 64% of doctors prescribed inhaled corticosteroids (ICS) and bronchodilators, 34% bronchodilators alone, and 2% ICS alone. The duration of bronchodilator therapy was 6–10 days for 51% of participants and 1–5 days for 44%. As regards the use of ICS for acute asthma attacks, 63% of the panel prescribed it, 16% sometimes, and 16% only if the child is taking ICS underlying. The use of nebulizers for treating acute episodes of wheezing or asthma was motley as 42% decided based on patient and family preference, 29% followed guidelines, 28% if the child is younger than three years, 18% always, 13% if the attack was severe, and 7% never. Also, the duration of ICS treatment in recurrent wheezing or suspected mild persistent asthma was various, as 29% prescribed them for 30–60 days, 26% for 7–14, 24% for 60–90 and 18% for 15–30. As regards the reason for nebulization as inhalation therapy in an acute wheezing/asthma episode, in 98% of cases was clinical (i.e., no response to treatment, unchanged or worsened clinical picture), in 52% history (i.e., comorbidity), in 19% functional (i.e., impaired spirometry), and in 7% biological (biomarkers). In addition, 85% think that rhinitis contributes to worsening asthma. Regarding the referral to a second/third level center, 57% of participants declared that the reason was for severe asthma attacks, 44% for difficulty in asthma management, 40% for children relapsing after ICS discontinuation, 28% for recurrent wheezing, 14% for any recurrent respiratory disease, 6% for chronic dry cough. The 95% of participants provided a written treatment plan for the patient for self-management of the wheezing/acute asthma attack. Consistently, 83% recommended early administration of systemic corticosteroids to children at significant risk of hospitalization or more severe forms. Moreover, 91% of pediatricians periodically reviewed children with asthma especially after each exacerbation, to modify the underlying therapy if necessary. As regards what device is recommended for recurrent wheezing/asthma to perform the background therapy, 60% prescribed pre-dosed spray with spacer always, 19% pre-dosed spray with spacer in children < one year old, 18% pre-dose spray with a spacer in children > 3 years, and 3% always nebulizer. The 89% of doctors increased background therapy for children who cough or wheeze during sport or other physical exertion and verified at each asthma visit (scheduled or urgent) the correct way of taking therapy using a spacer and pre-dosed spray. In addition, 93% had a spacer and spray in their practice to show the patient their proper use, and 91% believed having an app for families with the correct way to take medication was useful. Finally, 51% recommended specific immunotherapy to patients with asthma or allergic rhinitis, and 43% followed the advice suggested by the referral center, but 6% did not consider it.

## Discussion

The present survey reflected a cross-section of what is happening in Italy's real-world asthma management.

The results are substantially considerable and satisfying, even if some criticality exists. In particular, this survey was mainly completed by primary care pediatricians, such as 72% of the panel. As a result, the outcomes reflect what happens in the primary care setting. This aspect is important, as the primary care pediatrician should be the main figure directly involved in managing the child with asthma and interfacing with the family, so playing a pivotal role.

On the other hand, referral to a specialist center is essential to ensure a correct diagnosis, set up an appropriate and personalized treatment, and review the evolution of the therapy over time. In addition, the referral center can allow in-depth diagnosis using functional and biological examinations and prescribe biological drugs when necessary. Thus, integration between hospital and territory represents the primary way to ensure optimal care for children with asthma.

Furthermore, adherence to guidelines guarantees reliable and safe management and therapy. Therefore, the results obtained are substantially comforting and satisfactory.

In particular, the primary outcomes define a mirror of pediatric asthma management in Italy. Most pediatricians (63%) follow < 50 children with recurrent wheezing. This figure suggests an approximative prevalence of about 10%. In daily practice, the wheezing diagnosis is eminently clinical. There is a prevalent belief that asthma diagnosis is reliable from six years of age.

As concerns acute asthma/wheezing treatment, all pediatricians use inhalation therapy, prescribing prominently ICS associated with bronchodilators. However, one-third of participants prescribed only bronchodilators. This point should be stressed as the current recommendation is always combined therapy. There is, therefore, the need to implement the knowledge of adequate control of type 2 inflammation as a critical risk factor for severe asthma attacks, as recently underlined by GINA guidelines [4]. Regarding the duration of bronchodilator treatment, there is a dichotomous behavior, as half of pediatricians prescribe them for 5–10 days and 44% for 1–5 days. Similarly, ICS duration varies as 29% prescribe them for two months, 26% for one week, and 24% for two to three weeks. The correct duration of ICS therapy should be, according with GINA 2024 guidelines, a low dose ICS plus short acting beta_2_ agonist (SABA) as needed in the step 1 and daily low dose ICS plus as needed SABA for two or three months in step 2.

Nebulization choice is also various, and the main criteria regard patient preference and age, again with wide variations. Consistently, the pre-dose spray with a spacer is prescribed, considering heterogeneous reasons. These points should be clarified and require a better share by pediatricians. There is a need to implement the belief in the importance of adequate schedules and appropriate devices.

The need for deep analysis of asthma is based essentially on clinical criteria, including symptom worsening and increased relievers’ use.

There is a large consensus that rhinitis may worsen asthma. This reassuring outcome confirms that united airway disease is well-known and recognized. Namely, there is robust evidence that patients with rhinitis and asthma represent a more severe phenotype than patients with asthma or rhinitis alone [9]. Moreover, allergen-specific immunotherapy is well considered, as almost all participants favor its use.

Regarding asthma management, referrals to second/third-level centers depend on various reasons, but severe disease or treatment difficulty is the primary motivation.

Fortunately, most pediatricians ensure correct asthma management by providing a treatment plan, systemic corticosteroids as needed, step-on therapy, inhalation technique check, and spacer explanation.

Finally, most pediatricians believe that using apps to supervise asthma self-management is useful. In this regard, it is noteworthy to underscore that supervised self-management should be promoted as it assures optimal disease control and treatment adherence [10].

Therefore, the present survey emphasized that asthma management has its lights but also its shadows, although many aspects are in line with what the guidelines recommend.

Moreover, comparing some outcomes of the present survey with the previous study, it is comforting to learn that over the past five years, there has been an increased awareness of the proper management of asthma, especially about the use of controller drugs, i.e., ICSs, and their use whenever a bronchodilator is needed. In addition, there is also a more significant use of referral centers, which suggests better hospital-territory integration.

These outcomes are clinically relevant because they are derived from a large and representative panel of primary care and hospital pediatricians; thus, they mirror real-world practice. Conversely, asthma management could be further improved, and a closer partnership between primary care and the hospital is desirable. In addition, the strength of this initiative was the involvement of three scientific societies rooted mainly in Italy.

However, this survey had limitations, as it was impossible to calculate the exact rate of respondents. The invitation to participate was not personalized because a letter was not sent to each doctor. A banner appeared on each society's website inviting potential participants to the survey with a specific link to the platform. Therefore, it was impossible to have a precise idea of how many people actually read this invitation. Furthermore, many pediatricians could have been simultaneously enrolled in more than one of these three scientific societies.

In conclusion, the present survey confirmed that pediatric asthma management is relatively satisfactory, even if further improvement should concern a more widespread use of ICS for acute asthma/wheezing attacks, a better definition of duration of ICS and bronchodilators use, and hospital-primary care integration.

## Data Availability

All data generated and analyzed during this survey are included in this published article.

## Abbreviations

FIMP: Federazione Italiana Medici Pediatri
GINA: Global Initiative for Asthma
SABA: Short acting beta agonist
SIAIP: Società italiana di allergologia ed immunologia pediatrica
SIPPS: Società Italiana Pediatria Preventiva e Sociale
